# Negative Autoregulation by Fas Stabilizes Adult Erythropoiesis and Accelerates Its Stress Response

**DOI:** 10.1371/journal.pone.0021192

**Published:** 2011-07-08

**Authors:** Miroslav Koulnis, Ying Liu, Kelly Hallstrom, Merav Socolovsky

**Affiliations:** Department of Pediatrics and Department of Cancer Biology, University of Massachusetts Medical School, Worcester, Massachusetts, United States of America; University of Kansas Medical Center, United States of America

## Abstract

Erythropoiesis maintains a stable hematocrit and tissue oxygenation in the basal state, while mounting a stress response that accelerates red cell production in anemia, blood loss or high altitude. Thus, tissue hypoxia increases secretion of the hormone erythropoietin (Epo), stimulating an increase in erythroid progenitors and erythropoietic rate. Several cell divisions must elapse, however, before Epo-responsive progenitors mature into red cells. This inherent delay is expected to reduce the stability of erythropoiesis and to slow its response to stress. Here we identify a mechanism that helps to offset these effects. We recently showed that splenic early erythroblasts, ‘EryA’, negatively regulate their own survival by co-expressing the death receptor Fas, and its ligand, FasL. Here we studied mice mutant for either Fas or FasL, bred onto an immune-deficient background, in order to avoid an autoimmune syndrome associated with Fas deficiency. Mutant mice had a higher hematocrit, lower serum Epo, and an increased number of splenic erythroid progenitors, suggesting that Fas negatively regulates erythropoiesis at the level of the whole animal. In addition, Fas-mediated autoregulation stabilizes the size of the splenic early erythroblast pool, since mutant mice had a significantly more variable EryA pool than matched control mice. Unexpectedly, in spite of the loss of a negative regulator, the expansion of EryA and ProE progenitors in response to high Epo in vivo, as well as the increase in erythropoietic rate in mice injected with Epo or placed in a hypoxic environment, lagged significantly in the mutant mice. This suggests that Fas-mediated autoregulation accelerates the erythropoietic response to stress. Therefore, Fas-mediated negative autoregulation within splenic erythropoietic tissue optimizes key dynamic features in the operation of the erythropoietic network as a whole, helping to maintain erythroid homeostasis in the basal state, while accelerating the stress response.

## Introduction

**Figure 1:**
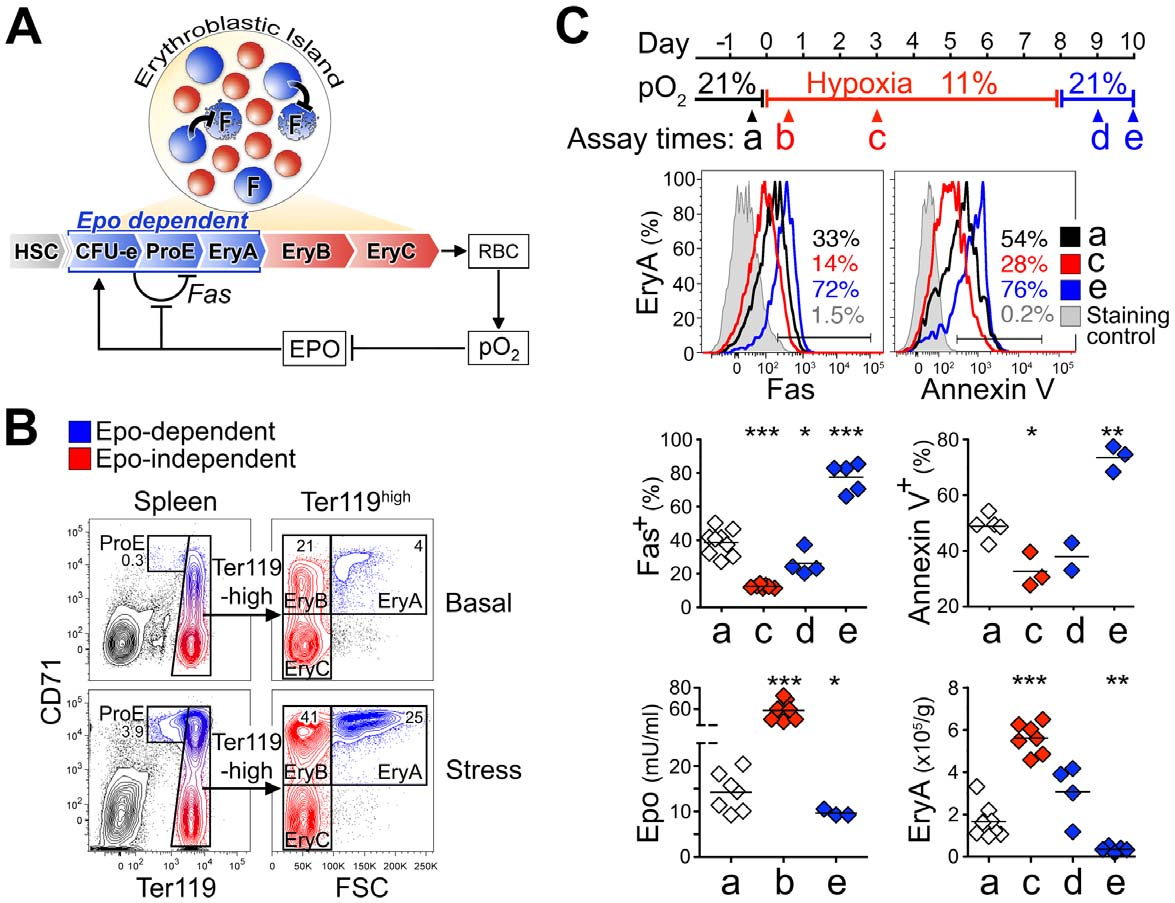
Epo regulation of erythropoiesis through Fas-mediated apoptosis. (**A**) Epo-dependent erythroblastic island precursors CFU-e, ProE and EryA (in blue) co-express Fas and FasL, and mature into Epo-independent EryB, EryC and red blood cells (RBC, in red). ‘F’  = Fas expressing cells, shown undergoing cell death as a result of interaction with FasL-expressing cells within the Epo- dependent (blue) compartment (black flat-headed arrow). A negative feedback loop driven by tissue pO_2_ regulates Epo levels in blood, which in turn enhance erythroblast survival, by either suppressing Fas and FasL expression, or by non-Fas dependent pathways. HSC  = hematopoietic stem cells. (**B**) Flow-cytometric identification of Epo-dependent ProE and EryA subsets (in blue) and Epo-independent EryB and EryC (in red), in adult Balb/C mouse spleen, in basal conditions (top panels) or 48 hours following Epo injection (300 U/ mouse, lower panels). ProE are defined as Ter119^med^CD71^high^ cells; Ter119^high^ cells are further subdivided based on forward scatter (FSC) and CD71 expression into EryA (CD71^high^Ter119^high^FSC^high^), EryB (CD71^high^Ter119^high^FSC^low^) and EryC (CD71^low^Ter119^high^FSC^low^). (**C**) The erythropoietic response of mice to a hypoxic environment. Mice (Balb/C) were examined either in the basal state (‘a’, 21% atmospheric oxygen), when housed in 11% oxygen for 8 days (assay times ‘b’ and ‘c’ at 13 hours and 3 days, respectively), and when placed back in normoxia (21%; assay times ‘d’ and ‘e’, at 1 and 2 days post-hypoxia). Top panels show representative flow-cytometric histograms of Fas expression and Annexin V binding at the indicated assay times. Gates refer to the Fas^+^ and Annexin V^+^ populations, determined with reference to staining controls in which either the anti-Fas antibody (left panel) or Annexin V (right panel) were omitted. The fraction of cells positive for Fas or Annexin V at each time point is noted. Middle panel shows a summary of similar data, 2 to 9 mice per time point. Lower panels show corresponding serum Epo levels and EryA cell number in spleen (expressed as total EryA cells /gram body weight). *p<0.05, **p<0.002, ***p<0.0001 (two-tailed *t* test, unequal variance).

The production of red blood cells (Erythropoiesis) is continuous throughout life, maintaining an optimal number of circulating red cells and tissue oxygen tension (pO_2_). A decrease in tissue pO_2_, as may occur in anemia, bleeding, high altitude or respiratory disease, drives erythropoiesis up to 10 fold its basal rate. This response is regulated through a negative feedback loop in which decreasing tissue pO_2_ increases synthesis of the hormone Epo ([1], [2], [3], Fig 1A). Epo-mediated activation of its receptor, EpoR [4], on erythroid progenitors increases their number and consequently, erythropoietic rate and tissue pO_2_. During accelerated rates of erythropoiesis, Epo cooperates with additional factors, notably glucocorticoid hormones and stem cell factor [5], [6], [7], [8].

Epo controls an early cellular compartment within the ‘erythroblastic island’, the developmental niche on the surface of a macrophage that supports erythroid maturation [9] (Fig 1A). Three to five cell divisions must elapse before cells in this early compartment mature into red blood cells. This inherent delay in the pO_2_/Epo-regulated feedback loop is likely to compromise the stability and rapid stress response of the erythropoietic system. Additional control mechanisms may therefore exist to compensate for this inherent delay.

The earliest Epo-dependent progenitor is the ‘Colony-forming Unit-erythroid’ or CFU-e, giving rise to colonies of at least 8 red cells within 48–72 hours *in vitro* [10]. Its erythroblast progeny are classified by their morphology [11]. We previously developed a flow-cytometric approach to identify erythroblasts directly in freshly-harvested mouse hematopoietic tissue, using cell surface markers CD71, Ter119 and cell size, measured by flow-cytometric forward scatter (FSC). We classify increasingly mature erythroid precursors subsets as ‘ProE’ (CD71^high^Ter119^med^), ‘EryA’ (CD71^high^Ter119^high^FSC^high^), ‘EryB’ (CD71^high^Ter119^high^FSC^low^) and ‘EryC’ (CD71^low^Ter119^high^FSC^low^)[12] (Fig 1A–B). We found that, in addition to the well documented increase in CFU-e [13], [14], the early erythroblast subsets ProE and EryA are responsive to EpoR signaling *in vivo* [12].

Epo promotes erythroblast survival *in vitro* [15], suggesting this mechanism may underlie its regulation of erythropoietic rate. Our recent experiments *in vivo* [12] confirm this hypothesis. During basal erythropoiesis, the majority of ProE and EryA undergo apoptosis, particularly in spleen, the murine organ of erythropoietic reserve [16]. During stress, high Epo decreases their apoptosis, increasing ProE and EryA number [12]. The reasons for this apparently wasteful mechanism of erythropoietic rate regulation have not been addressed experimentally.

EpoR activates several survival pathways, including Stat5-mediated induction of bcl-x_L_ [17], [18], [19], [20], other Stat5 targets [21], [22], [23], [24] and the EpoR-activated phosphoinositide 3-kinase (PI3K) -AKT pathway [25], [26]. The death receptor Fas, and its ligand, FasL, were first proposed to contribute to erythroid homeostasis based on their expression in human bone-marrow [27]. We found that, *in vivo* in the mouse, splenic ProE and EryA, but not their bone-marrow counterparts, co-express Fas and FasL. During stress, high Epo suppresses their Fas expression, strongly correlating with their decreased apoptosis [12]. These findings suggested the hypothesis that, in the basal state, splenic ProE and EryA negatively regulate their own survival through Fas and FasL-mediated inter-cellular interactions; and that Epo-mediated Fas suppression is a key mechanism regulating erythropoietic expansion during stress (Fig 1A) [12].

Negative autoregulation through co-expression of Fas and FasL was previously implicated in terminating the clonal expansion of activated T cells [28], [29], [30]. Similarly, we recently found that Fas and FasL co-expression in fetal liver erythroid progenitors terminates their initial wave of expansion at the onset of fetal erythropoiesis [31]. Here we set out to assess the contribution of Fas and FasL to erythropoiesis in the adult. We bred *lpr* or *gld* mice, mutant in Fas and FasL respectively, onto the Rag1^−/−^ immune-deficient background, in order to avoid an autoimmune syndrome that may impact erythropoiesis. Our findings in both the *lpr*-Rag1^−/−^ and *gld*-Rag1^−/−^ mice were similar, showing that Fas-mediated autoregulation in spleen negatively regulates erythropoiesis at the whole animal level. Strikingly, these experiments also revealed that Fas-mediated autoregulation imparted key dynamic properties to the erythropoietic system. We found that it is responsible for stabilizing the basal precursor pool, enhancing their resistance to random perturbations. Further, it also accelerated the erythropoietic response to high Epo, an unexpected effect for a negative regulator. Therefore, the dynamic properties of the Fas-mediated autoregulation offset the dynamic deficits of the slower, pO_2_/Epo-regulated feedback. Of interest, negative autoregulatory loops, in the context of simple transcriptional networks, were shown to accelerate the response to a stimulus, and to enhance network stability [32], [33], [34], [35]. Our findings here suggest that these loops, which are abundant in biological systems, similarly improve the homeostasis and dynamic responses of lineage-specific progenitors *in vivo*.

## Materials and Methods

Please also see Text S1: Supplementary Material and Methods section.

### Mice

B6.MRL-*Fas^lpr^*/J (Jackson Laboratories stock # 000482) were crossed with B6.129S7-*Rag1^tm1Mom^*/J (stock # 002216) to obtain homozygous double-mutant *lpr*-Rag1^−/−^ mice (C57BL/6J background). The homozygous double mutant *gld*-Rag1^−/−^ mice (Balb/C background) were obtained by crossing CPt.C3-*Fasl^gld^*/J (Jackson Laboratories, stock # 002932) with C.129S7(B6)-*Rag1^tm1Mom^*/J (Jackson Laboratories stock # 003145). Other Balb/C mice for the hypoxia chamber experiments were purchased from Taconic. All experiments were approved by the University of Massachusetts Medical School IACUC committee, under protocol number A1586.

#### Flow cytometry

Flow cytometry was performed as described [12] on freshly isolated spleen and bone-marrow cells. Dead cells were excluded using DAPI (Roche, Indianapolis, IN) or 7-AAD (BD Biosciences) viability dye. Data were analyzed using FlowJo software (Tree Star, Ashland, OR).

#### Statistical analyses

Statistical analyses was performed using PASW (SPSS, Chicago, IL) and SAS (SAS, Cary, NC) statistical software. Data was log-transformed and analyzed by general linear mixed model ANOVA with gender and genotype as fixed effects and experiment as the random effect. Unadjusted p-values were reported in the figures for each gender-genotype group.

## Results

### Hypoxia and return to normoxia alter Fas expression and survival of early erythroblasts

Erythropoietic stress results in suppression of Fas expression and reduced apoptosis in splenic ProE and EryA [12]. These findings suggested the model illustrated in Figure 1A, in which Fas-mediated cell death is a result of intercellular interactions between Fas and FasL-co-expressing cells within the spleen early erythroblast compartment; these interactions are blocked by high Epo during stress (Fig 1B).

Here we investigated this model further by housing mice in a hypoxic environment of 11% oxygen for 8 days, followed by a return to normoxia (21% oxygen). Hypoxia caused a sharp increase in plasma Epo (Fig 1C, lower left panel), suppressing Fas expression in EryA and enhancing their survival (Fig 1C, upper panel: example of flow-cytometric measurements of Fas expression and Annexin V binding; middle panels: summary of data form 2 to 9 mice per time point). Decreased apoptosis and Fas expression were associated with increased spleen EryA (Fig 1C, lower right panel). A return to 21% oxygen resulted in a rapid reversal, with Epo and EryA declining below their starting levels, and both Fas expression and apoptosis rising well above their starting basal levels. These results suggest that modulation of Fas-mediated apoptosis by Epo plays a role in both the expansion of the EryA pool in response to hypoxia, and in its rapid contraction with the return to normoxia.

### Effect of reducing Fas-mediated apoptosis with Fas:Fc *in vivo*

To examine the effect of Fas on EryA survival directly, we administered MyD88^−/−^ mice with the purified chimeric molecule, Fas:Fc [36], which acts as a decoy receptor, binding FasL on the surface of EryA and blocking its ability to activate Fas. Control MyD88^−/−^ mice were injected intraperitoneally with an equal volume of saline. The MyD88^−/−^ strain was used in order to avoid potential reaction to contaminating bacterial Lipopolysaccharide (LPS) in the Fas:Fc preparation [37]. A single administration of Fas:Fc (100 µg) resulted in a 20% reduction in unoccupied surface FasL in both splenic ProE and EryA, measured by binding of the Fas-blocking monoclonal antibody MFL3, directed against FasL [38] (not shown). This procedure decreased the number of Annexin V^+^ EryA from 70% in control to 50% in Fas:Fc -injected mice by 48 hours. There was an associated increase in EryA, and a doubling in blood reticulocytes (Fig S1). Reticulocytes, identified by their RNA content, mature within 24 hours [39]; a doubling of reticulocytes suggests a doubling of erythropoietic rate over the most recent 24 hours. These results support a causal relationship between Fas expression, EryA survival and erythropoietic rate.

### The dose/response characteristics of Epo-mediated Fas suppression and EryA expansion

**Figure 2:**
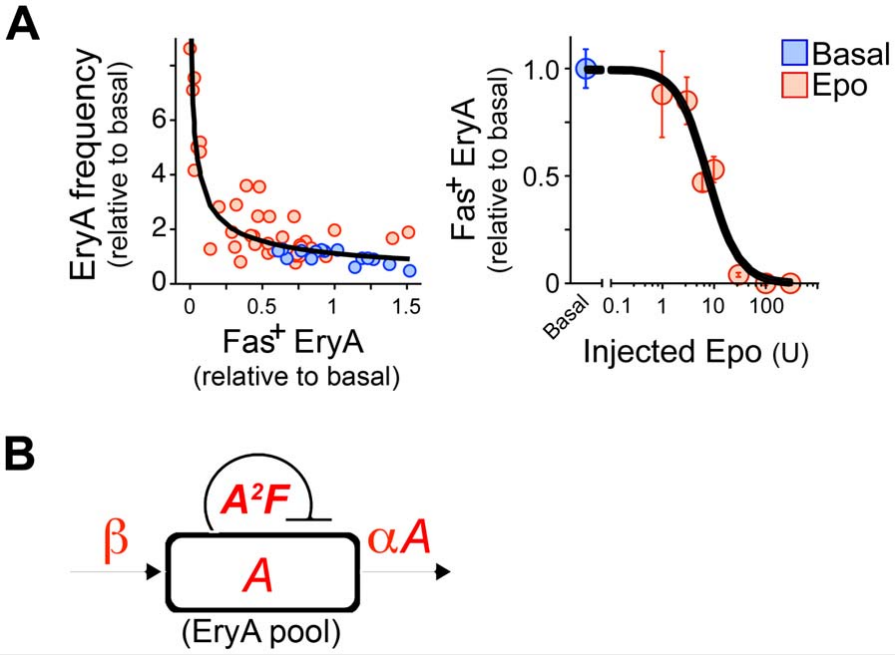
Fas and FasL-mediated negative autoregulation of the EryA pool. (**A**) Wild-type Balb/C mice were injected with a single Epo injection subcutaneously, of 1, 3, 6, 10, 30, 100 or 300 U/ 25 g body weight. Spleen EryA were examined on day 3 post injection. *Left panel* shows EryA cell frequency relative to basal frequency, plotted against the number of EryA cells that express Fas (Fas^+^ EryA, expressed as a ratio to basal levels). Data points represent individual mice. Blue  = mice in the basal state (n = 15), red  = mice injected with Epo (n = 38). For clarity only mice injected with 30 U (which maximally suppress Fas) or less are included in the left panel. Data is fitted with a curve derived from the mathematical model described in panel (**B**) and in the Text S2. *Right panel* shows the dependence of Fas^+^ EryA on the dose of injected Epo, in the same dataset as in the left panel; all mice injected with a given Epo dose were pooled into one data point, mean ± sem. (**B**) Schematic of the factors that regulate the size of the EryA pool, ‘*A’*, measured as the fraction of all Ter119^+^ cells that are EryA. *F*  = fraction of EryA cells that express Fas. ß  = input into the EryA pool from earlier progenitor stages. α*A*  = output from the EryA pool into later erythroid subsets (EryB). *A^2^F*  = Fas-mediated cell loss. See mathematical model described in Text S2. In panel (**A**) of this figure, *A* is plotted against *F*, expressed as a ratio to the *A* and *F* values in the basal state, respectively.

To assess the quantitative relationship between Epo, Fas suppression and EryA numbers we injected mice (n = 38) with varying doses of Epo, of between 1 and 300 Units /25 g body weight. We measured the Fas response on day 3, previously found to correspond to the lowest Fas expression level attained following acute Epo injection [12]. We found that EryA frequency in Ter119^+^ spleen cells was inversely related to the fraction of EryA cells that expressed Fas (Fig 2A, left panel). Half maximal suppression of Fas expression was seen in mice injected with 10 U/25 g (Fig 2A, right panel), corresponding to a doubling of EryA frequency. Complete suppression of Fas expression was seen at 30 U/ 25 g, and resulted in a 6-fold increase in EryA frequency.

### The frequency of EryA is inversely related to their Fas expression

The relationship between EryA frequency, and the fraction of EryA that express Fas, may be fitted by a model in which EryA undergo Fas-mediated negative autoregulation (Fig 2B). We considered the frequency of EryA within the erythroblastic island, ‘*A*’, to be the result of three principal factors: first, a continuous input from earlier progenitors, ‘*ß’*; second, a continuous output, proportional to *A,’αA’*, into more differentiated progenitor subsets; and last, Fas-dependent cell death. We assumed that Fas-mediated cell loss would result when two EryA cells, expressing Fas and FasL respectively, interact within the erythroid compartment. The probability of such an encounter is proportional to the product of the frequencies of Fas^+^ and FasL^+^ EryA cells. This product is approximately equal to *A^2^F*, where ‘*F*’ denote the fraction of EryA cells that express Fas (Fig 2B; the fraction of EryA that express FasL in adult spleen is high enough that it can be approximated to 1; see Text S2: ‘Regulation of the EryA progenitor pool by Fas’). These considerations allow the steady-state level of EryA, at any given level of Fas, to be found by solving a quadratic equation. The inverse relationship between EryA and Fas in Fig 2A is fitted well by a hyperbolic curve that represents the (positive) steady-state solutions for EryA (‘*A*’) in this equation, at different steady-state levels of Fas *(‘F*’) (R^2^ = 0.89, Text S2). This goodness of fit supports the model’s key assumption, that Fas-mediated cell loss is a result of negative autoregulation within the EryA pool, and is proportional to the square of the frequency of EryA within the erythroblastic island. Further, it suggests that Epo concentration, which sets the desired steady-state EryA pool size, does so in part by regulating the level of Fas expression in the EryA population (Fig 1A).

Of note, using a different modeling approach, we found a similar relationship in fetal liver, where Fas-mediated loss of early erythroblasts was proportional to the square of the frequency of early erythroblasts in the tissue [31].

### Generation of Fas and FasL-deficient mice on an immune-deficient background

We made use of the *lpr* and *gld* mouse strains that carry naturally-occurring loss-of-function mutations of Fas and FasL, respectively [40]. The *lpr* mutation consists of an insertion of an early transposable element, carrying a polyadenylation signal, in the second intron of the Fas gene. This causes premature termination of the transcript and a drastic decrease in Fas transcription, though it does not fully eliminate it. In the *gld* strain, there is a point mutation at the C-terminus of FasL that abolishes its ability to bind Fas. Both the *lpr* and *gld* mice develop a lymphoid proliferative autoimmune syndrome [41]. In order to avoid this complication, we bred these mouse strains onto an immune-deficient, Rag1^−/−^ background that lacks T and B cells [42]. Erythroid parameters, such as basal hemoglobin concentration, though consistent within a given inbred strain, differ somewhat between mice of different genetic backgrounds, likely reflecting quantitative differences in the control of erythropoiesis [43]. We therefore chose to generate two distinct genetic background strains. The *gld*-Rag1^−/−^ mice were bred on the Balb/C background, and the *lpr*-Rag1^−/−^ mice on the C57BL/6 background. As controls, we used age and strain-matched Rag1^−/−^ mice, which on either the Balb/C or the C57BL/6 backgrounds, have normal erythropoietic parameters when compared with wild-type mice of the same background strain. Further, the double homozygous mutant strains, *gld*-Rag1^−/−^ and *lpr*-Rag1^−/−^, showed no sign of autoimmunity, as evident from their small spleen size, absence of immune cells from the blood and spleen, and lack of anemia (Table S1 and data not shown).

### Basal Erythropoiesis in *lpr*-Rag1^−/−^ and *gld*-Rag1^−/−^ mice

**Figure 3:**
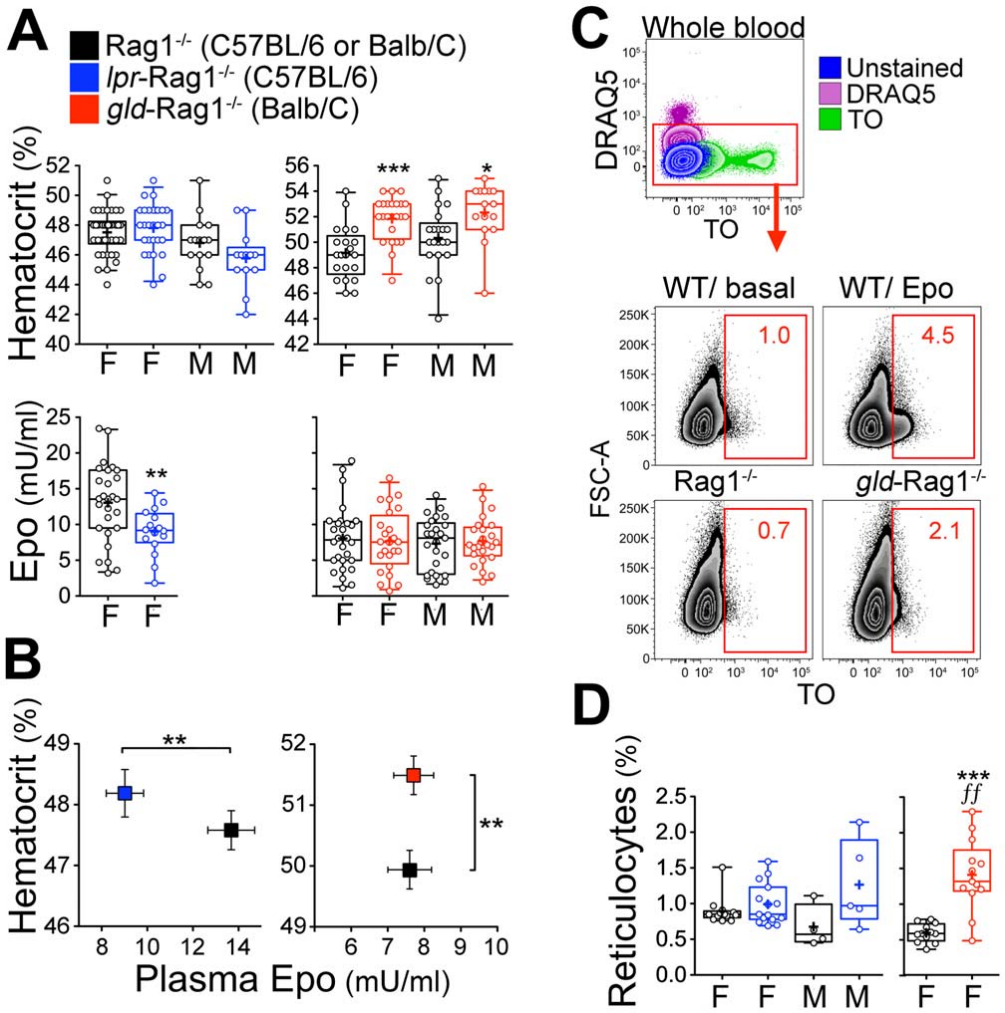
Increased erythropoiesis in mice deficient in the Fas pathway. Legend in **A** also applies to panels **B**, **D**. *lpr*-Rag1^−/−^ mice are on the C57BL/6 background (in blue), and are compared with control Rag1^−/−^ mice on the C57BL/6 background. *gld*-Rag1^−/−^ mice are on the Balb/C background (in red), and are compared with control Rag1^−/−^ mice on the Balb/C background. (**A**) Hematocrit ( = fraction of the blood volume that is due to red cells) and Plasma Epo of *lpr*-Rag1^−/−^, *gld*-Rag1^−/−^ and Rag1^−/−^ age and strain-matched control mice. M =  males. F =  females. Box and whiskers delineate the central 50% and 90% of readings, respectively. Median is indicated with a horizontal line; arithmetic mean with a ‘+’. Data points correspond to individual mice. Between 11 and 40 mice examined per genotype. *p<0.05, **p<.005, ***p<0005 (ANOVA). (**B**) Hematocrit *vs.* plasma Epo in the subset of mice where both values were measured, in the basal state, for *lpr*-Rag1^−/−^ and matched Rag1^−/−^ control mice (left panel), and for *gld*-Rag1^−/−^ and matched Rag1^−/−^ controls (right panel). Data are mean ± sem of ≥16 mice *p≤0.001 (two-tailed *t* test, unequal variance). (**C**) Flow cytometric measurement of reticulocyte number. Top: whole blood stained with either DRAQ5 (detects DNA) or thiazole orange (TO, detects both DNA and RNA). Reticulocytes lack a nucleus but retain RNA. They therefore form a DRAQ5-negative, TO-positive population. Bottom panel shows analysis in wild-type (WT) mice either in the basal state or following Epo injection; and in *gld*-Rag1^−/−^ and control Rag1^−/−^ mice. (**D**) Reticulocyte in *lpr*-Rag1^−/−^, *gld*-Rag1^−/−^ and matched Rag1^−/−^ controls, measured by flow-cytometry ***p<0.0001, two-tailed t-test, unequal variance; *ff = * p<0.001, F test.

The chronic absence of an erythropoietic regulator may not be apparent from simple inspection of the hematocrit in the steady state [20]. This is due to a vast erythropoietic reserve, coupled with the pO_2_/Epo negative feedback loop which automatically adjusts Epo levels and erythropoietic rate so as to maintain a near- normal tissue pO_2_. Therefore, to assess how the loss of a regulator affects steady state erythropoiesis requires analysis at all levels of the pO_2_/Epo negative feedback loop, including Epo concentration and the erythroid progenitor and precursor pools. We first examined the hematocrit (the fraction of all blood volume that is attributable to red blood cells), blood reticulocytes and plasma Epo. *lpr*-Rag1^−/−^ mice on the C57BL/6 background had a normal reticulocyte count and normal hematocrit (Figure 3A, top left panel, Table S1). However, plasma Epo was significantly lower, by 35%, than in control mice (p = 0.001; Epo =  9.0±0.8 mU/ml, mean ±sem, in *lpr*-Rag1^−/−^, and 13.7±1.0 for Rag1^−/−^ controls; Fig 3A, bottom left panel, and Fig 3B). Therefore, *lpr*-Rag1^−/−^ mice compensate for the chronic absence of a negative regulator through the pO_2_/Epo negative feedback loop, decreasing Epo concentration so as to avoid an unnecessary increase in hematocrit.

By contrast, the *gld*-Rag1^−/−^ mice, on the Balb/C background, had a significantly elevated hematocrit (p<0.00001; hematocrit =  52.0±0.3% *vs*. 49.8±0.4% for *gld*-Rag1^−/−^ *vs.* Rag1^−/−^ respectively, mean±sem, Figures 3A; a similar difference was found in a second group of mice assayed using a Coulter counter, Table S1). Reticulocyte count was also more than double that of controls (p<0.0001; Reticulocyte count =  1.40±0.14% *vs*. 0.59±0.04% for *gld*-Rag1^−/−^ *vs.* Rag1^−/−^ respectively, Fig 3C–D), and hemoglobin concentration was also significantly elevated in 2 independent experiments (Table S1). As comparison, mice we housed for 3 weeks in 12% oxygen, equivalent to an altitude of 14,000 feet, increased their hematocrit from 51.6±0.2 to 57.8±0.5%, an increase of 6%. Therefore, the 2.2% increase in hematocrit in the *gld*-Rag1^−/−^ mice represents approximately a third of the erythropoietic output in high altitude hypoxia. Unlike the *lpr*-Rag1^−/−^, plasma Epo in the *gld*-Rag1^−/−^ mice was not significantly different to that of controls (Fig 3A), possibly because of the already low basal Epo in control Balb/C mice which was ∼40% lower than in control mice on the C57BL/6 background (Fig 3A, lower panels, black symbols).

Of note, responses by both *gld*-Rag1^−/−^ (Balb/C background) and the *lpr*-Rag1^−/−^ (C57BL/6 background) mice have in common a higher erythropoietic rate per unit plasma Epo than in matched control mice, consistent with the absence of a negative regulator of erythropoiesis.

### Increased splenic CFU-e, ProE and EryA in *gld*-Rag1^−/−^ and *lpr*-Rag1^−/−^ mice

**Figure 4:**
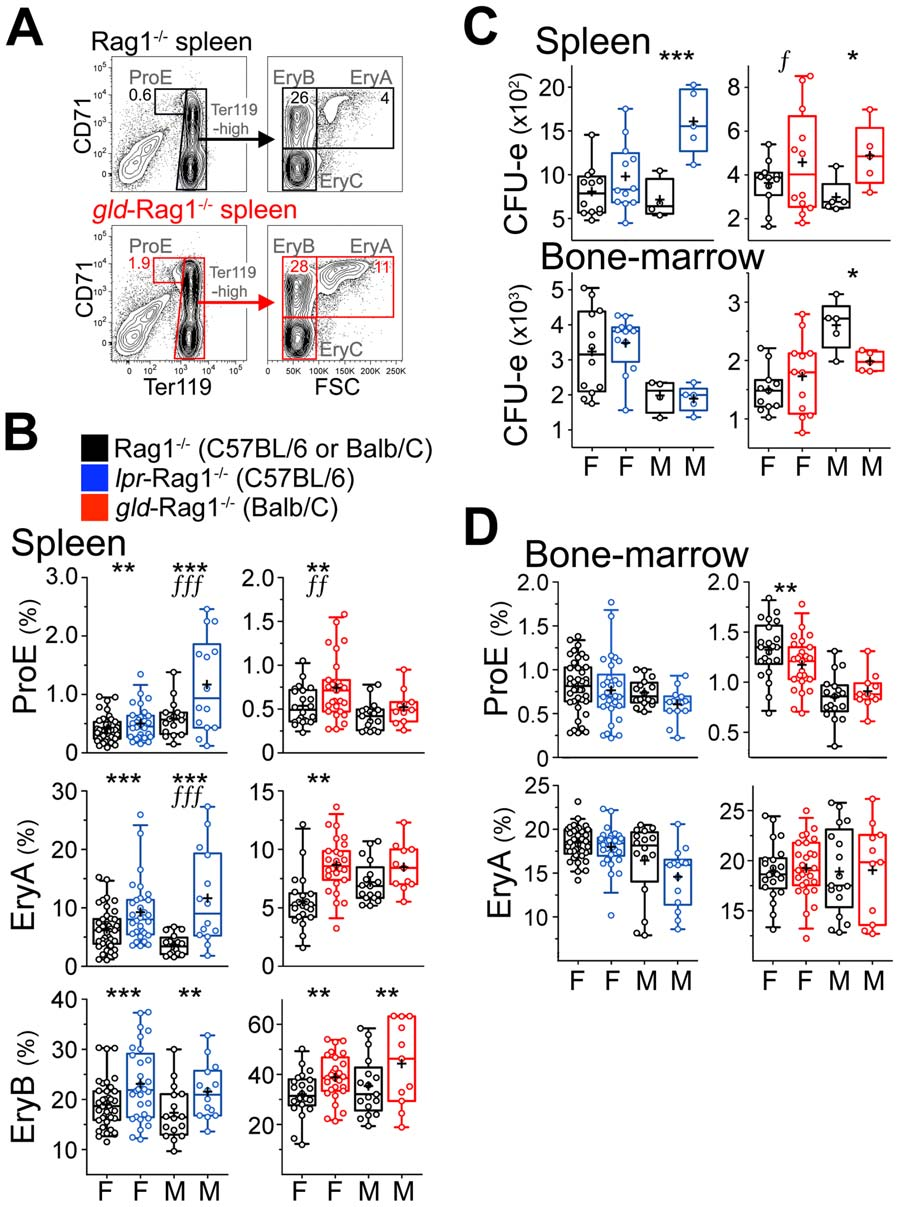
Increased frequency of spleen, but not bone-marrow, erythroid progenitors and precursors in mice deficient in the Fas pathway. The Legend in panel B also applies to C,D. (**A**) Representative flow-cytometric analysis of spleen erythroid subsets in *gld*-Rag1^−/−^ and matched Rag1^−/−^ controls, showing increased frequency of ProE and EryA within Ter119^+^ cells. (**B**) Frequency of erythroblast subsets in spleen erythropoietic tissue, measured as in **A**, expressed as fraction of all spleen Ter119^+^ cells. F = female M = male. Box and whiskers delineate the central 50% and 95% of readings, respectively, with the median indicated with a horizontal line and arithmetic mean with a ‘+’. Data points are individual mice (11–40 mice per sex/genotype combination). Data was pooled from several independent experiments. (**C**) CFU-e progenitors in spleen and bone-marrow. Data pooled from two independent experiments for females, and one experiment for males, for each genotype. (**D**) Frequency of erythroblast subsets in bone-marrow expressed as fraction of all spleen Ter119^+^ cells. For all panels, *p<0.05 **p<0.02, ***p<0.002 (ANOVA, for difference in means). *f* = p<0.05 ,*ff* = p<0.02, *fff* = p<0.002 (F test, for difference in variance).

**Figure 5:**
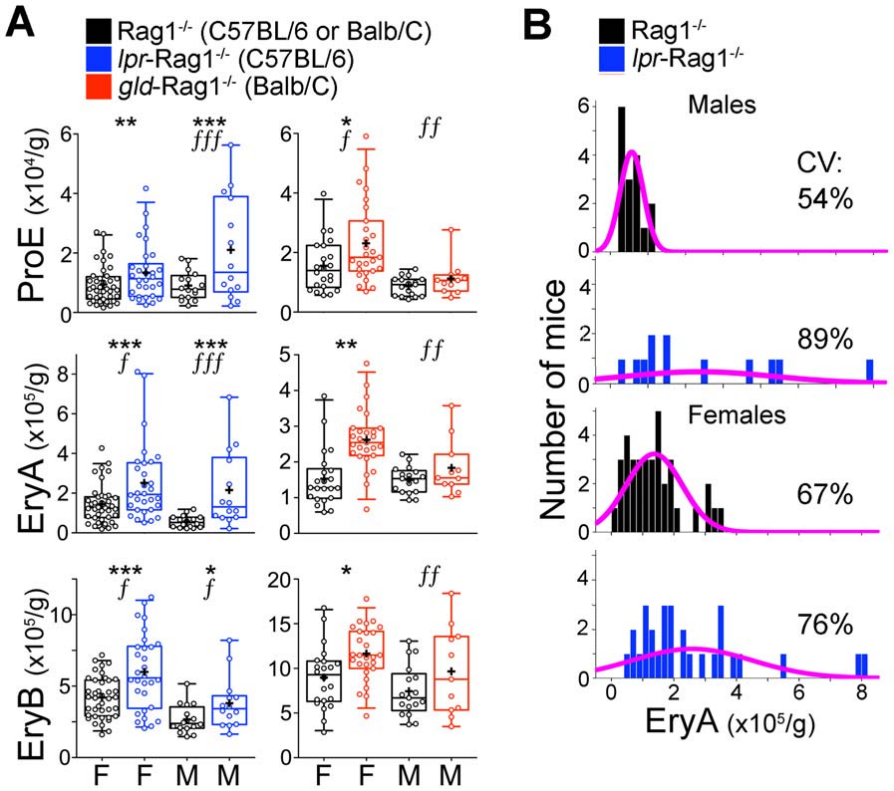
Loss of Fas function results in a larger and more variable basal spleen erythroid progenitor pool. (**A**) Spleen erythroid subsets ProE, EryA or EryB, expressed as absolute number of cells per gram body weight, in *gld*-Rag1^−/−^, *lpr*-Rag1^−/−^ and matched Rag1^−/−^ controls, shown separately for male (M) and female (F) mice. Data corresponds to the same mouse dataset as in Fig 4. Box and whiskers delineate the central 50% and 90% of readings, respectively, with the median indicated with a horizontal line and arithmetic mean with a ‘+’. Data points correspond to individual mice. Between 11 and 40 mice examined per genotype; data pooled from several independent experiments. *p<0.05, **p<0.005, ***p<0.0005 (ANOVA, for difference in means); *f* = p<0.05, *ff* = p<0.005, *fff* = p<0.0005 (F test, for difference in variance). (**B**) Frequency distribution histograms for EryA, in male and female *lpr*-Rag1^−/−^ and matched Rag1^−/−^ controls. The coefficient of variation for each group is shown. Purple line is the fitted normal distribution curve. Same data set as in panel (**A**).

A representative flow-cytometric histogram in Figure 4A shows that the frequency of the ProE and EryA subsets within *gld*-Rag1^−/−^ spleen erythropoietic tissue (Ter119^+^ cells) was increased. A similar analysis in 11 to 40 mice per strain/sex combination is summarized in Figures 4B and 4D. An increase in subset frequency does not necessarily reflect a corresponding increase in cell number, since it may also arise as a result of decreased cell number in other subsets. We therefore also examined the absolute number of cells in each of the erythroid precursor subsets, computed by multiplying the subset frequency data by the fraction of Ter119^+^ cells per spleen and the spleen weight, for each individual mouse (Fig 5A).

These data show significant increases, ranging from 1.5 to 4-fold, depending on genotype and sex, in both the frequency and absolute number of spleen CFU-e, ProE, EryA and EryB, in *gld*-Rag1^−/−^ and *lpr*-Rag1^−/−^ mice (Figures 4A–C and 5A). Specifically, all the splenic ProE, EryA and EryB subsets showed increased frequency within the Ter119^high^ compartment in both *gld*-Rag1^−/−^ and *lpr*-Rag1^−/−^ mice relative to matched controls, with this increase reaching statistical significance in ten of the twelve comparisons made, namely ProE (p<0.02), EryA (p<0.002) and EryB (p<0.002) in female *lpr*-Rag1^−/−^ mice, ProE (p<0.002), EryA (p<0.002) and EryB (p<0.02) in male *lpr*-Rag1^−/−^ mice, ProE (p<0.02), EryA (p<0.02) and EryB (p<0.02) in female *gld*-Rag1^−/−^ mice, and EryB (p<0.02) in male *gld*-Rag1^−/−^ mice. The frequency of ProE and EryA in male *gld*-Rag1^−/−^ mice was also increased relative to controls but did not reach statistical significance. Similarly, the absolute number of cells in each of these erythroid subsets increased in both *gld*-Rag1^−/−^ and *lpr*-Rag1^−/−^ mice, reaching significance in nine of the twelve comparisons made, namely, ProE (p<0.005), EryA (p<0.0005) and EryB (p<0.0005) in female *lpr*-Rag1^−/−^ mice, ProE (p<0.0005), EryA (p<0.0005) and EryB (p<0.05) in male *lpr*-Rag1^−/−^ mice, and ProE (p<0.05), EryA (p<0.005) and EryB (p<0.05) in female *gld*-Rag1^−/−^ mice. The number of ProE, EryA and EryB in male *gld*-Rag1^−/−^ mice also increased but the differences did not reach statistical significance (Fig 5A). The number of *gld*-Rag1^−/−^ mice available for analysis was smaller than that of the other sex/strain combinations (Fig 4B, 5C), possibly accounting for the failure of the change in their erythroblast subsets to reach statistical significance.

Of note, there was no significant change in any of the equivalent subsets in bone-marrow (Fig 4D) for any sex/strain combination. These results are consistent with the pattern of erythroid Fas and FasL co-expression, which is largely restricted to spleen [12]. The largest increases were observed in ProE and EryA, in agreement with their higher Fas and FasL expression [12].

### Variability in basal erythropoietic rate and in the size of spelnic erythroid subsets in *gld*-Rag1^−/−^ and *lpr*-Rag1^−/−^ mice

Whilst the mean size of the ProE and EryA pools increased in *gld*-Rag1^−/−^ and *lpr*-Rag1^−/−^ mouse populations, their actual size in individual mice was highly variable. Using an F-test we found significantly higher variance in both the frequency and absolute number of nearly all erythroblast subsets in the *gld*-Rag1^−/−^ and *lpr*-Rag1^−/−^ mice, and in *gld*-Rag1^−/−^ reticulocytes, compared with matched Rag1^−/−^ controls (significantly different variance is marked with an ‘*f*’; Figures 3D, 4B–C, 5A). The EryA frequency distributions (Figures 5B and S2) show the larger spread of EryA pool size in the mutant mice. We also found increased coefficient of variation (CV) for EryA and ProE in male, but less so in female, *gld*-Rag1^−/−^ and *lpr*-Rag1^−/−^ mice (Figures 5B and S2). The coefficient of variation measures variability independently of the population mean. Overall, CV values for all splenic erythroblast subsets and for peripheral blood reticulocytes in both *gld*-Rag1^−/−^ and *lpr*-Rag1^−/−^ male and female mice, are significantly increased (p = 0.017, Figure S2 B). These findings indicate that a key function of Fas-mediated negative autoregulation is to suppress variability in the steady-state precursor pool and in erythropoietic rate, thus stabilizing the basal state.

### A delayed response to Epo-driven erythropoietic stress in *gld*-Rag1^−/−^ and *lpr*-Rag1^−/−^ mice

**Figure 6:**
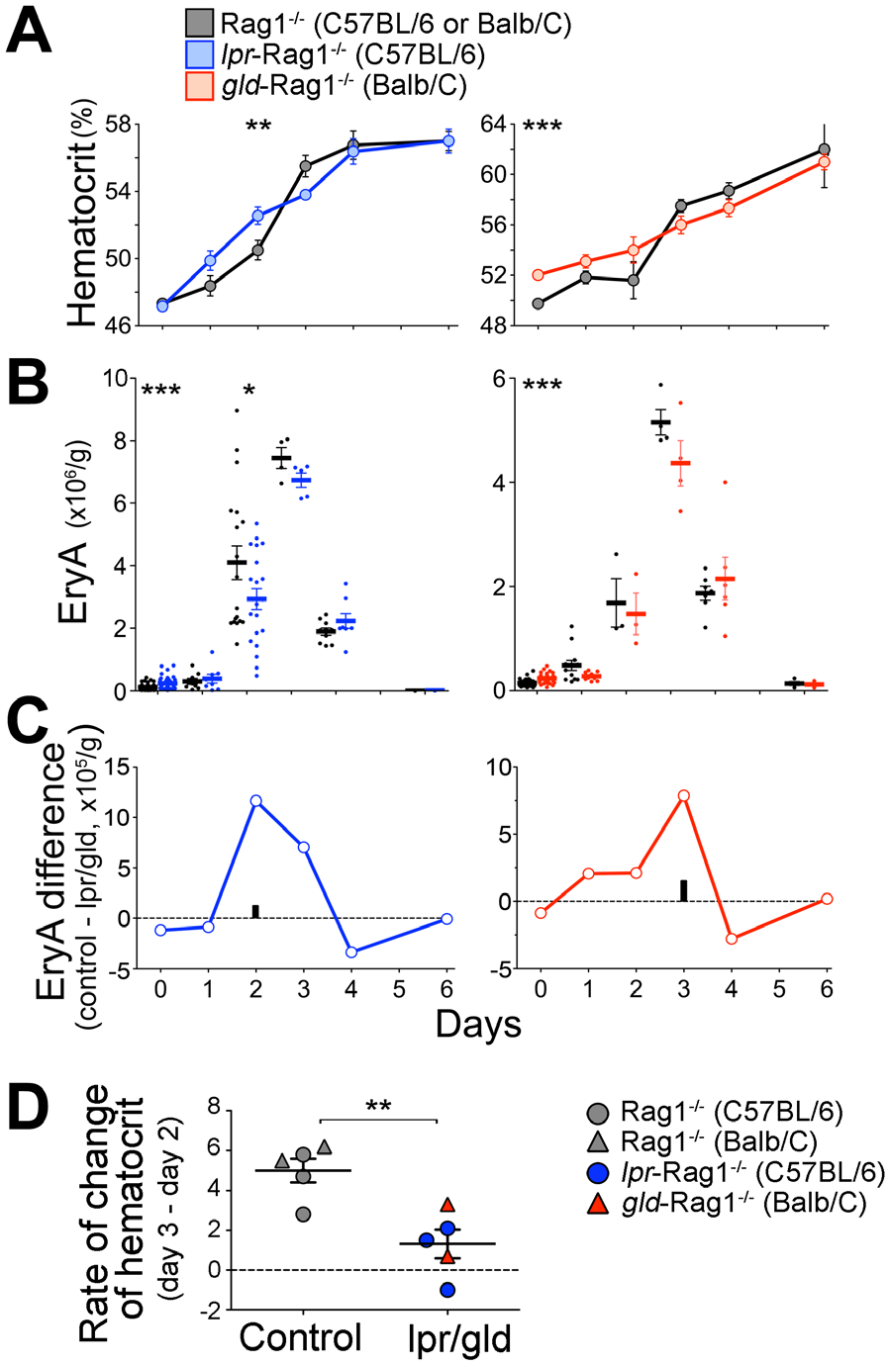
Delayed response to Epo-induced stress in mice deficient in the Fas pathway. (**A–D**) *gld*-Rag1^−/−^, *lpr*-Rag1^−/−^ and matched Rag1^−/−^ control mice were injected with Epo (300 U/ 25 g body weight) subcutaneously at t = 0. The erythropoietic response was followed for 6 days. Data is mean ± sem for 3 to 18 mice per time point per genotype, pooled from up to 3 experiments per time point. Data at t = 0 is the basal state data shown in Figures 3A and 5A, pooled for males and females. (**A**) Hematocrit measurements (**B**) Spleen EryA (cells per gram body weight) in the same mouse set as in the top panel. Data points are individual mice, with the mean ±sem for each day marked as a horizontal line. (**D**) The difference in mean EryA number (shown in panel **B**) between the mutant *gld*-Rag1^−/−^ or *lpr*-Rag1^−/−^ and their matched Rag1^−/−^ controls, for each day. The size of the corresponding control (Rag1^−/−^) EryA pool is marked with a black bar. For all panels: *p<0.05, **p<0.005, ***p<0.0005 (*t* test, unequal variance). (**D**) The rate of change in hematocrit between days 2 and 3 following Epo injection. The same dataset as in panel (A), showing the differences in hematocrit measured in multiple independent experiments on days 2 and 3. Altogether 5 independent comparisons are shown. For all panels: *p<0.05, **p<0.005, ***p<0.0005 (t test, unequal variance).

We took two approaches to assess the stress response of the *gld*-Rag1^−/−^ and *lpr*-Rag1^−/−^ mice. First, we injected mice with a single high dose of Epo (300 U/ 25 g body weight), and followed the resulting increase in erythropoietic rate for 6 days (Fig 6A–D). Initially, on days 1 and/or 2, hematocrit was higher in both *gld*-Rag1^−/−^ and *lpr*-Rag1^−/−^ mice compared with controls, by 1–2%, possibly reflecting the larger basal erythroblast pool in these mice. However, between days 2 and 3, there was a significantly faster increase in hematocrit in control mice, which rose by over 4% in the space of 24 hours, overtaking the hematocrit of *gld*-Rag1^−/−^ and *lpr*-Rag1^−/−^ mice (Fig 6A, 6D). There was no equivalent rapid increase in hematocrit in the *gld*-Rag1^−/−^ and *lpr*-Rag1^−/−^ mice. By subtracting the mean hematocrit on day 2 from the mean hematocrit on day 3, in 5 independent comparisons between independent experiments on those days, we found that the rate of change in the hematocrit was significantly higher in control mice (p<0.005, Fig 6D). The slower increase in hematocrit in both *gld*-Rag1^−/−^ and *lpr*-Rag1^−/−^ mice at this time was due to a slower expansion of EryA precursors, in spite of their larger basal EryA pools (Fig 6B, 6C). The difference in EryA expansion was largest on days 2 (*lpr*-Rag1^−/−^ mice) and 3 *(gld*-Rag1^−/−^ mice) (Fig 6C), corresponding to the time when Epo-mediated Fas suppression in wild-type mice reaches its peak [12].

Of note, EryA expansion in control mice was a massive, 30 to 60 fold increase over the basal EryA pool. The shortfall in EryA on day 2 in the *lpr-*Rag1^−/−^ mice was equivalent to 10 times the size of the basal EryA pool, or 30% of the total expansion in control mice on that day (Figure 6B,C, p<0.05; the size of the basal EryA pool is marked Fig 6C as a black bar). A similar delay in EryA expansion was seen in *gld*-Rag1^−/−^ mice (Figure 6B,C). Further, injection of a much lower Epo dose (10 U/ 25 g body weight) in *lpr*-Rag1^−/−^ mice again resulted in delayed hematocrit and ProE responses (Fig S3). Therefore, the presence of Fas-mediated negative autoregulation accelerates the erythropoietic response over a wide Epo stress range.

**Figure 7:**
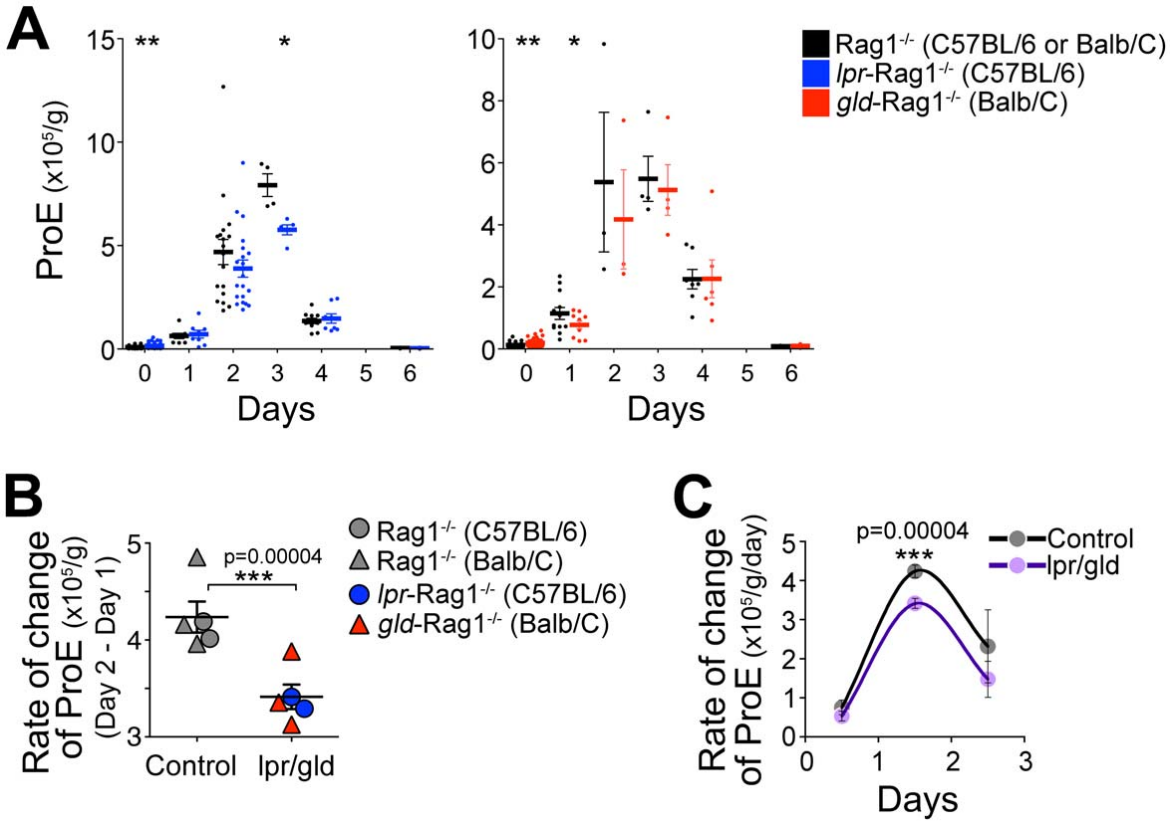
Delayed response to Epo-induced stress in mice deficient in the Fas pathway. (**A–C**) Analysis of the ProE response to Epo injection. The same experiment and dataset as in Figure 6. (**A**) Spleen ProE (cells per gram body weight). Data points are individual mice, with the mean ±sem for each day marked as a horizontal line. Data pooled from 1 to 3 experiments with 3 to 18 mice per genotype. (**B**) The rate of change in spleen ProE between days 1 and 2 post-Epo injection in the mutant *gld*-Rag1^−/−^ (indicated in red) or *lpr*-Rag1^−/−^ (blue) and their matched Rag1^−/−^ controls. Dataset as in panel (A). Each point represents the mean difference in ProE between independent experiments done on days 2 and 3, in 5 independent comparisons. (**C**) The rate of change in spleen ProE throughout the first 3 days of response to Epo, computed as in panel B. The rate of change between days 0 and 1, days 1 and 2, and days 2 and 3, were plotted on days 0.5, 1.5 and 2.5, respectively. Data points represent pooled mutant (*lpr/gld*) or control differences (mean ±sem). For all panels: *p<0.05, **p<0.005, ***p<0.0005 (*t* test, unequal variance).

Analysis of changes in the ProE population showed similar results. In spite of its larger size in the basal state, expansion of the ProE pool in both *gld*-Rag1^−/−^ and *lpr*-Rag1^−/−^ mice was slower between days 1 and 3, the differences in the absolute size of the pools reaching significance on days 1 (*gld*-Rag1^−/−^ mice) and 3 *(lpr*-Rag1^−/−^ mice) (Fig 7A). Furthermore, the rate of increase in ProE in response to Epo peaked in all mice between days 1 and 2, attaining a significantly lower level in *gld*-Rag1^−/−^ and *lpr*-Rag1^−/−^ mice compared with matched controls (p = 0.00004, Fig 7 B,C).

### A delayed erythropoietic stress response to reduced atmospheric oxygen in *gld*-Rag1^−/−^ mice

**Figure 8:**
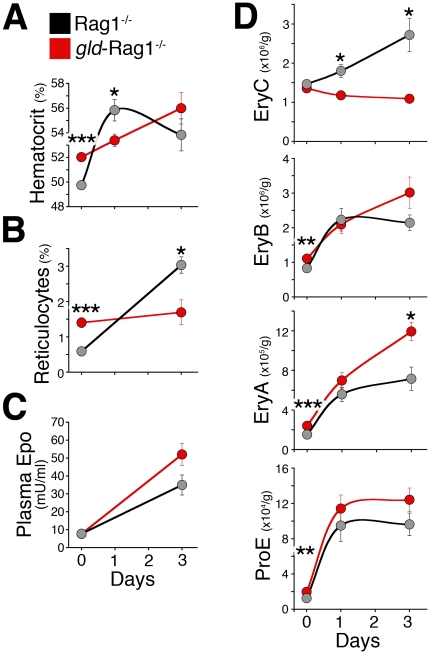
Delayed response to hypoxia-induced stress in mice deficient in the Fas pathway. (**A–D**) *gld*-Rag1^−/−^ or control mice were transferred to a hypoxia chamber with ambient 11% oxygen for 1 or 3 days. Data is mean ±sem for 4 to 9 age and gender-matched mice per time point per genotype. Data at t = 0 is the basal state data shown in Figures 3A and 5A, pooled for males and females. (**A**) Hematocrit measurements, performed via CritSpin microcentrifugation. (**B**) Reticulocyte measurements, performed flow cytometrically, as in Figure 3C. (**C**) Plasma Epo, measured by ELISA. (**D**) Spleen erythroid subsets ProE, EryA, EryB and EryC, expressed as absolute number of cells per gram body weight in *gld*-Rag1^−*/*−^ and matched Rag1^−/−^ controls. For all panels: *p<0.05, **p<0.005, ***p<0.0005 (*t* test, unequal variance).

In a second approach, we examined the response of the *gld*-Rag1^−/−^ mice to an acute reduction of atmospheric oxygen to 11% (Fig 8). The response to reduced atmospheric oxygen is complex since, in addition to elevating serum Epo, it stimulates additional cytokines as well as changes in ventilation and plasma volume that may indirectly alter erythropoietic responses [8], [44], [45]. Furthermore, the actual increase in serum Epo in response to lower atmospheric oxygen is determined not only by the initial hypoxic stimulus, but also by the ensuing erythropoietic response, which determines the duration of tissue hypoxia (Fig 1A). We chose to examine *gld*-Rag1^−/−^ mice, since, unlike the *lpr*-Rag1^−/−^ strain, their starting baseline Epo levels are very similar to those of their matched controls (Fig 3B). We found that in spite of their higher starting hematocrit and reticulocyte count, the increase in erythropoietic rate in *gld*-Rag1^−/−^ mice was significantly slower, as seen by a significantly delayed increase in hematocrit (Fig 8A) and a lower reticulocyte count (Fig 8B). There was a correspondingly slower increase in EryC erythroblasts (Fig 8D), at both 24 and 72 hours. This sluggish response presumably prolonged the tissue hypoxia in these mice, as reflected by their higher serum Epo at 72 hours (Fig 8C). These results clearly show a delayed stress response in mice lacking Fas-mediated negative autoregulation of erythroblasts.

Unlike the response to injection of a fixed, high Epo dose (Fig 6), the slower erythropoietic response to hypoxia in the *gld*-Rag1^−/−^ mice was not associated with a difference in the size of the ProE/ EryA pools at the early (24 hour) time point. Such a difference may have occurred earlier; alternatively, hypoxia may accelerate the maturation of EryA cells, so that reserve, Fas^+^ EryA cells in control mice do not contribute to an increase in the EryA pool but instead differentiate rapidly and contribute to the increase that we see in the EryC pool by 24 hours (Fig 8D). Indeed, it has been observed previously that hypoxic stress as a result of bleeding or phehylhydrazine stress accelerates erythroblast maturation [46], [47], [48]. The larger number of EryA in *gld*-Rag1^−/−^ by 72 hours presumably reflects the higher Epo level at that time, in turn a result of the more prolonged stress these mice presumably experience, due to their sluggish erythropoietic response to the initial hypoxic stimulus.

## Discussion

We investigated the role of Fas and FasL-mediated negative autoregulation in the early erythroblast pool. We generated *gld*-Rag1^−/−^ and *lpr*-Rag1^−/−^ mouse strains, deficient in FasL and Fas, respectively, bred onto two distinct genetic backgrounds. Both these strains showed similar erythropoietic deficits. They confirm the hypothesis that Fas and FasL are negative regulators of splenic EryA and ProE, resulting in negative regulation of erythropoiesis at the whole animal level. In addition, they show a striking, non-redundant role for Fas in stabilizing basal erythropoiesis. Surprisingly, in spite of the removal of a negative regulator and the consequent larger basal precursor pool, the response of the mutant mice to erythropoietic stress was significantly delayed. These findings reveal that an autoregulatory loop local to erythropoietic tissue can exert key dynamic properties on erythropoiesis as a whole. They also provide experimental evidence that regulation of erythroid precursors through apoptosis, though apparently wasteful, accelerates the response to erythropoietic stress.

### The presence of a local negative autoregulatory loop in spleen erythropoietic tissue

Several lines of evidence suggest that EryA and ProE negatively regulate their own survival through their Fas and FasL-mediated interactions. First, the pattern of co-expression of both Fas and FasL by EryA and ProE [12], [31]; second, the close apposition of cells within the erythroblastic island, making an interaction between Fas^+^ and FasL^+^ cells possible [31]; and last, our finding that a mathematical model in which EryA negatively regulate their own survival via the Fas/FasL interaction, accounts well for the experimental data correlating splenic EryA frequency with their Fas expression, across a wide range of Epo concentrations *in vivo* (Fig 2 and Text S2).

### Fas-mediated negative autoregulation decreases erythropoiesis at the whole-animal level

Both acute and chronic inhibition of Fas suggest that it negatively regulates erythropoiesis at the level of the whole animal. An acute decrease in erythroblast FasL by transient administration of the decoy receptor Fas:Fc resulted in an acute increase in erythropoietic rate, reducing ProE and EryA apoptosis and doubling reticulocyte number by 48–72 hours (Fig S1).

Chronic loss of Fas function in the *gld*-Rag1^−/−^ and *lpr*-Rag1^−/−^ mice is likely to elicit compensation through the pO_2_/Epo-mediated negative feedback loop, which automatically adjusts Epo levels and erythropoietic rate so as to maintain a near-normal basal hematocrit and tissue pO_2_. Alhough *lpr*-Rag1^−/−^ mice had normal reticulocytes and hematocrit, their plasma Epo was significantly lower, by 35%, than in matched Rag1^−/−^ controls, evidence of compensatory adjustment. By contrast, *gld*-Rag1^−/−^ mice were apparently unable to significantly lower their already low plasma Epo. Consequently, their reticulocyte numbers in peripheral blood, a direct measure of erythropoietic rate, more than doubled, and there was a corresponding significant 2.2% increase in hematocrit. This increase is equivalent to a third of the increase in hematocrit we observed in mice housed in 12% oxygen, equivalent to hypoxia at 14,000 feet.

While many pathways have been implicated in the regulation of erythropoietic rate, a specific contribution to the stress response *in vivo* had been determined for very few. Of note, mice lacking ERK1, a recently described negative regulator of splenic erythropoiesis, showed a similar increase in hematocrit to that seen here for the *gld*-Rag1^−/−^ mice, without a significant change in plasma Epo [49]. Taken together, both the *lpr*-Rag1^−/−^ and *gld*-Rag1^−/−^ strains show increased erythropoietic rate per unit plasma Epo when compared with matched controls, confirming that local negative regulation at the level of splenic erythropoietic tissue has a negative effect at the level of the whole animal.

There is no indication that Fas and FasL interact with alternative receptors or ligands. Therefore, the difference in the response of the *gld*-Rag1^−/−^ and *lpr*-Rag1^−/−^ mice at the level of the hematocrit and serum Epo is likely attributable to their different genetic backgrounds. The response of both these mouse strains at the level of the ProE and EryA precursors, the direct sites of action of Fas and FasL, was very similar.

### Loss of Fas function results in a specific increase of spleen ProE and EryA pools

We found a significant 1.5 to 4 fold increase in the number of splenic, but not bone-marrow, ProE and EryA in both the *gld*-Rag1^−/−^ and *lpr*-Rag1^−/−^ mice, consistent with the pattern of erythroid Fas and FasL expression[12]. We also identified an increase in CFU-e specific to spleen, suggesting that these cells are regulated by Fas, in agreement with their counterpart in fetal liver [31]. Bone marrow progenitor subsets were either normal or even decreased in number, possibly as a compensatory response to their increase in spleen (Fig 4C,D).

The increase in spleen ProE, EryA and CFU-e is due to the absence of a spleen-specific negative regulator, rather than erythropoietic stress, since it is not associated with elevated serum Epo, and since there is no associated increase of bone-marrow progenitors. It is unlikely that the increase in ProE/EryA is a consequence of expansion in an earlier, Fas-regulated progenitor compartment, since there was no significant change in bone-marrow erythroid progenitors, platelets or white cells (Table S1). An earlier report of increased CFU-S in adult *lpr* and *gld* mice [50] is complicated by the autoimmune syndrome in these mice, which in the present work we addressed by breeding the *lpr* and *gld* mutant mice onto the immune-deficient Rag1^−/−^ background.

The expansion in ProE and EryA precursor pools in the *gld*-Rag1^−/−^ and *lpr*-Rag1^−/−^ mice represents a substantial non- redundant negative regulatory function of Fas. Nevertheless, it is likely to be an underestimate of the actual number of ProE/EryA that are regulated by Fas in wild-type mice, as suggested by the response to stress (see below). Compensatory mechanisms that could ameliorate the absence of Fas or FasL include upregulation of alternative negative regulators such as ERK1 [49], attenuation of alternative EpoR anti-apoptotic pathways such as Stat5-induced bcl-x_L_, phosphoinositol-3 kinase/AKT and suppression of Bim and Foxo3a [17], [24], [51], or a decrease in factors that stimulate erythropoiesis such as BMP [52].

### A stabilizing function for the Fas and FasL-mediated negative autoregulatory loop

Random variation in the number of progenitors is an inevitable consequence of inherent fluctuations in biological systems [53], [54], [55], found in mice that are genetically identical, of similar age and housed in similar stable conditions. The pO_2_/Epo-mediated negative feedback loop (Fig 1A) adjusts overall mean erythropoietic rate in the face of such fluctuations over time, but the intrinsic delay in this loop may result in oscillations and limit the system's stability. Here we found that in the absence of Fas or FasL, there was a significant increase in variability, reflected by both increased variance and increased coefficient of variation, of splenic ProE, EryA and EryB in individual mice, compared with control mice (Fig 4B, Fig 5A). Bone-marrow erythroid subsets were not affected, but there was increased variability of overall erythropoietic rate, as reflected by the reticulocyte counts. Therefore, Fas-mediated autoregulation in spleen has a stabilizing effect on erythropoiesis at the whole animal level, offsetting the limitations of the pO_2_/Epo-mediated negative feedback loop.

The stabilizing influence of the Fas/FasL interaction is a result of its sensitivity to the size of the erythroblast pool. For comparison, a recently identified cell-autonomous negative regulator of splenic erythropoiesis *in vivo*, ERK1, does not appear to contribute to the stability of erythropoiesis [49]. Fas -mediated apoptosis of ProE or EryA depends on the probability that two cells expressing Fas and FasL respectively, encounter each other within the erythropoietic niche. This probability is dependent on their frequency in tissue (Fig 2 and Text S2 *).* Should their frequency be in excess, Fas-mediated loss of EryA would accelerate, providing an automatic correction. Conversely, a shortage of EryA would lower the probability of their interaction and death, allowing their number to increase. Both negative and positive corrections take place locally in erythropoietic tissue, with little delay and without the need to engage the pO_2_/Epo-mediated negative feedback loop, avoiding potentially deleterious corrective swings in systemic tissue pO_2_.

### Fas-mediated Negative autoregulation accelerates the stress response

Unexpectedly for mice lacking a negative erythropoietic regulator, the response of *gld*-Rag1^−/−^ and *lpr*-Rag1^−/−^ mice to acute Epo administration was delayed. The splenic EryA pool expanded 30 fold its basal size in control mice by day 2; this massive increase was reduced by 30% in the *gld*-Rag1^−/−^ and *lpr*-Rag1^−/−^ mice, reflecting a significant shortfall, equivalent to 10 times the size of the basal EryA pool. This shortfall occurred in spite of a larger than normal basal EryA pool in the mutant mice. We found a similar delay in the expansion of ProE cells in the *gld*-Rag1^−/−^ and *lpr*-Rag1^−/−^ mice.

**Figure 9:**
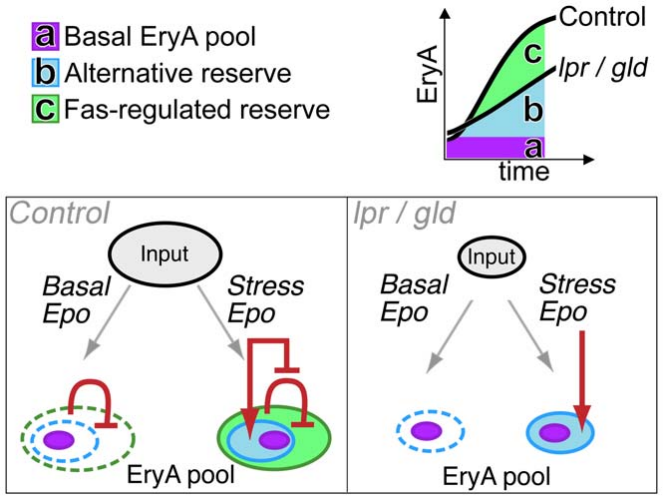
Absence of a Fas-regulated EryA reserve delays the response to stress. EryA cells are continuously formed from earlier precursors (‘input’). In the basal state, when Epo concentrations are low, only a small fraction of these cells survive, forming the ‘basal EryA pool’ (in purple). The remaining EryA undergo apoptosis, either through Fas (‘Fas-regulated reserve’, green) or alternative mechanisms (‘Alternative reserve’, blue). Together, the EryA reserve pools are 30 to 60 fold the size of the basal pool (see Figure 6). During the initial response to stress, high Epo levels rescue the EryA reserve pools from apoptosis, resulting in an immediate increase in the size of the surviving EryA pool and an increase in erythropoietic rate (solid colors indicate surviving cells, dashed lines indicate cells that underwent apoptosis). We suggest that *lpr* and *gld* mice partially compensate for the absence of the Fas-regulated reserve by generating fewer EryA cells (a smaller input). In this way, the absence of Fas –mediated apoptosis does not excessively increase the basal EryA pool (which does increase 1.5–4 fold, see Figure 5; this increase is much smaller than the stress-induced increased and is not shown). During stress, the absence of the Fas-regulated reserve in *lpr* and *gld* mice reduces the number of EryA that may be immediately recruited into the surviving EryA pool and consequently delays the stress response.

To explain this phenomenon, we propose the model illustrated in Figure 9. EryA are continuously formed from earlier progenitors ( = EryA input) but the majority dies, with only a small fraction remaining as the basal EryA pool. However, the apoptosis-prone EryA form a reserve population that may be rapidly recruited by high Epo during stress. We propose that the rapid, 30-fold expansion in EryA within 2 days of Epo administration corresponds to the size of the reserve EryA population. The smaller initial expansion in the *gld*-Rag1^−/−^ and *lpr*-Rag1^−/−^ mice suggests that part of the EryA reserve is regulated through Fas, and is missing in these mutant mice.

These observations suggest that the high apoptotic rates of ProE and EryA in the basal state, in part due to Fas, provide a mechanism that accelerates the stress response. The rescue of ProE/EryA from apoptosis allows a faster increase in their number compared with their generation by cell division from earlier progenitors. This faster increase accelerates erythropoietic rate and prevents a prolonged tissue hypoxia. We found that the response of the erythropoietic system to reduced atmospheric oxygen was similarly delayed in *gld*-Rag1^−/−^ mice, as reflected by lower reticulocyte counts and a slower increase in hematocrit. In this more complex, hypoxic stimulus to the system, we noted that the principal difference in the erythroblast pools between *gld*-Rag1^−/−^ and control mice appeared at the EryC stage, rather than in the earlier, ProE and EryA stages. We suggest that this may be due to accelerated maturation of erythroblasts, noted in the older literature in response to hypoxic stress [46], [47], [48].

Of note, in ERK1−/− mice, the stress response is timely and somewhat amplified [49]. Unlike Fas-mediated autoregulation, which is exerted at the level of erythroblasts, ERK1 appears to suppress a much earlier, BMP-dependent BFU-e progenitor, which is therefore unlikely to contribute to the initial, acute phase of stress.

### The negative autoregulatory motif

Negative autoregulation is a frequent motif in biological networks. Computational and experimental approaches in simple transcriptional networks in E.Coli suggested that it has two principal effects: conferring resistance to random fluctuations, and accelerating the response to a stimulus [32], [33], [34], [35], [56], [57]. To our knowledge, the functional role of negative autoregulation within higher-level intercellular networks had not been tested experimentally. Our work suggests that the negative regulatory motif may exert similar ‘logic’ in higher-level networks, helping to maintain both stability and a fast stress response of tissue progenitors.

## Supporting Information

Figure S1 Associated with Fig 1: Inhibition of Fas with Fas:Fc decreases EryA death and increases erythropoietic rate. MyD88^−/−^ mice (C57BL/6 background) were each injected intraperitoneally with 100 µg human purified Fas:Fc chimeric protein (BD Biosciences), or with an equal volume of saline. **(A)** Flow-cytometric histogram of Annexin V binding of spleen EryA cells, showing decreased apoptosis 48 hours following injection of Fas:Fc. **(B)** Summary of Annexin V binding in two independent experiments, at 48 hours and at 72 hours post-injection. Data points correspond to individual mice. **(C)** Summary of reticulocyte count (red blood cells younger than 24 hr, identifiable by their cytoplasmic RNA, which is absent in older red cells) in the same mice/experiments as in panel (B). Fas:Fc caused an increase in reticulocytes, reflecting increased erythropoietic rate.
(TIF)

Figure S2 Associated with Fig 5: Increased variance in *lpr/gld* erythroid progenitor subsets. **(A)** Frequency distribution histograms for EryA, in male and female *gld*-Rag1^−/−^ and matched Rag1^−/−^ controls. The coefficient of variation for each group is shown. Purple line is the corresponding normal distribution curve. Same data set as in Fig 3D. A similar analysis for the *lpr*-Rag1^−/−^ mice is shown in Figure 3E. **(B)** Coefficient of variation (CV) for subsets ProE, EryA–C and retics in male or female *lpr*-Rag1^−/−^ or and *gld*-Rag1^−/−^ mice and corresponding Rag1^−/−^ controls. The difference in CV between control and *lpr/gld* is significant at p = 0.017 (paired t test, treating the CV as a standard statistical variable).
(TIF)

Figure S3 Associated with Fig 6: Delayed response to Epo injection in *lpr*-Rag1^−/−^ mice. Female mice were injected with 10 U of Epo subcutaneously. Hematocrit, ProE and EryA progenitors were measured on day 3 post injection. *p<0.05 (two-tailed t test, unequal variance). Basal hematocrit values are for *lpr*-Rag1^−/−^ females.
(TIF)

Table S1 Associated with Fig 3: Complete blood counts (CBC) for the indicated mouse strains. Number of mice used for each strain in each experiment is indicated in parentheses. HCT  = hematocrit. HGB  = hemoglobin. RBC  = red blood cells. MCV  = mean corpuscular volume. WBC  = white blood cells. PLT  = platelets. All data are mean ±sem. *t* test (unequal variance) p values are indicated.
(TIF)

Text S1 Supplementary Materials and Methods.
(DOCX)

Text S2 Regulation of the EryA progenitor pool by Fas.
(DOCX)
