# Supplementary figures and images for: Negative Autoregulation by Fas Stabilizes Adult Erythropoiesis and Accelerates Its Stress Response
Source: PLoS One. 2011 Jul 8;6(7):e21192. doi: 10.1371/journal.pone.0021192 (PMC3132744; doi:10.1371/journal.pone.0021192)

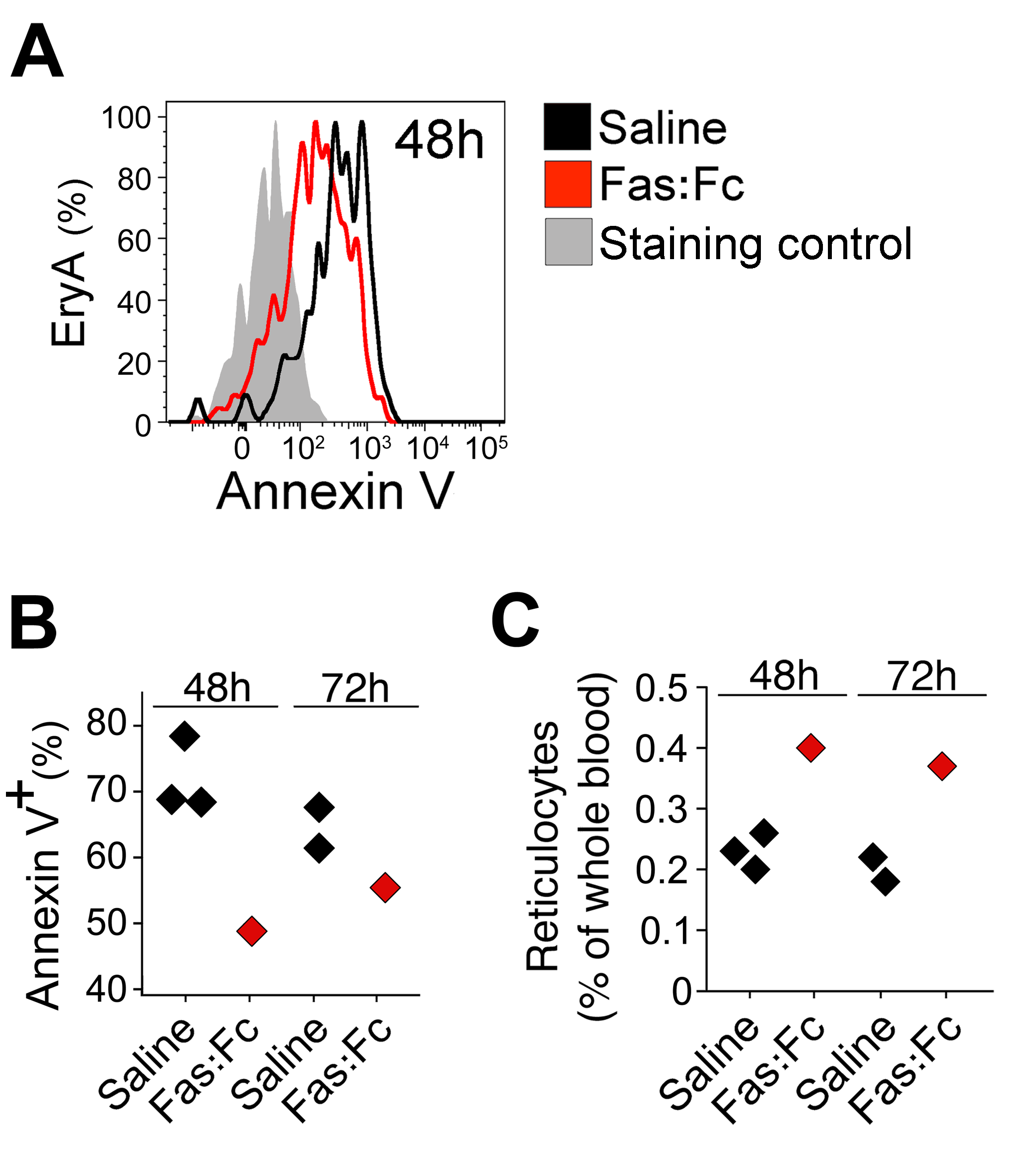

Supplement: Figure S1 — Associated with Fig 1: Inhibition of Fas with Fas:Fc decreases EryA death and increases erythropoietic rate. MyD88−/− mice (C57BL/6 background) were each injected intraperitoneally with 100 µg human purified Fas:Fc chimeric protein (BD Biosciences), or with an equal volume of saline. (A) Flow-cytometric histogram of Annexin V binding of spleen EryA cells, showing decreased apoptosis 48 hours following injection of Fas:Fc. (B) Summary of Annexin V binding in two independent experiments, at 48 hours and at 72 hours post-injection. Data points correspond to individual mice. (C) Summary of reticulocyte count (red blood cells younger than 24 hr, identifiable by their cytoplasmic RNA, which is absent in older red cells) in the same mice/experiments as in panel (B). Fas:Fc caused an increase in reticulocytes, reflecting increased erythropoietic rate. (TIF) [file pone.0021192.s001.tif]

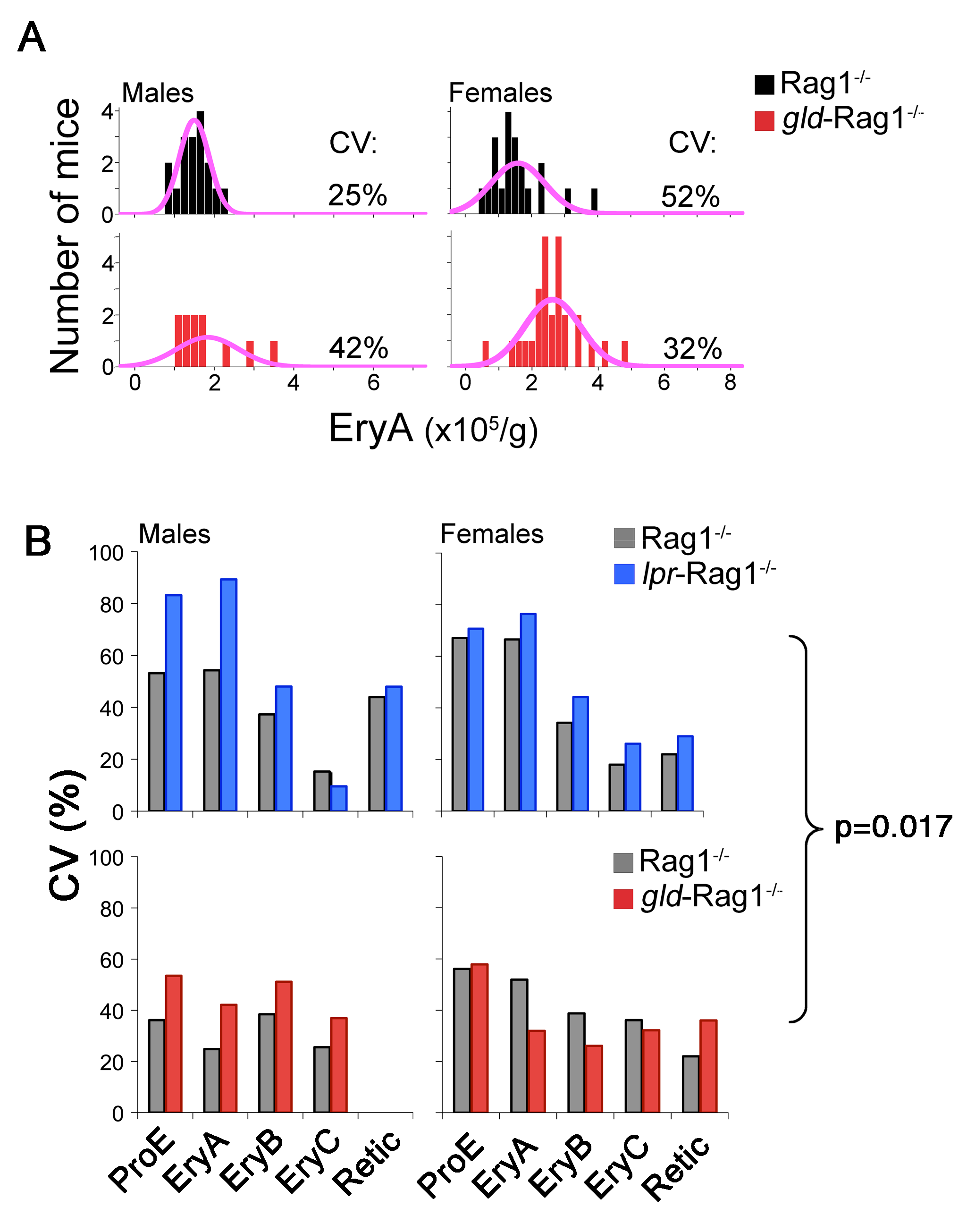

Supplement: Figure S2 — Associated with Fig 5: Increased variance in lpr/gld erythroid progenitor subsets. (A) Frequency distribution histograms for EryA, in male and female gld-Rag1−/− and matched Rag1−/− controls. The coefficient of variation for each group is shown. Purple line is the corresponding normal distribution curve. Same data set as in Fig 3D. A similar analysis for the lpr-Rag1−/− mice is shown in Figure 3E. (B) Coefficient of variation (CV) for subsets ProE, EryA–C and retics in male or female lpr-Rag1−/− or and gld-Rag1−/− mice and corresponding Rag1−/− controls. The difference in CV between control and lpr/gld is significant at p = 0.017 (paired t test, treating the CV as a standard statistical variable). (TIF) [file pone.0021192.s002.tif]

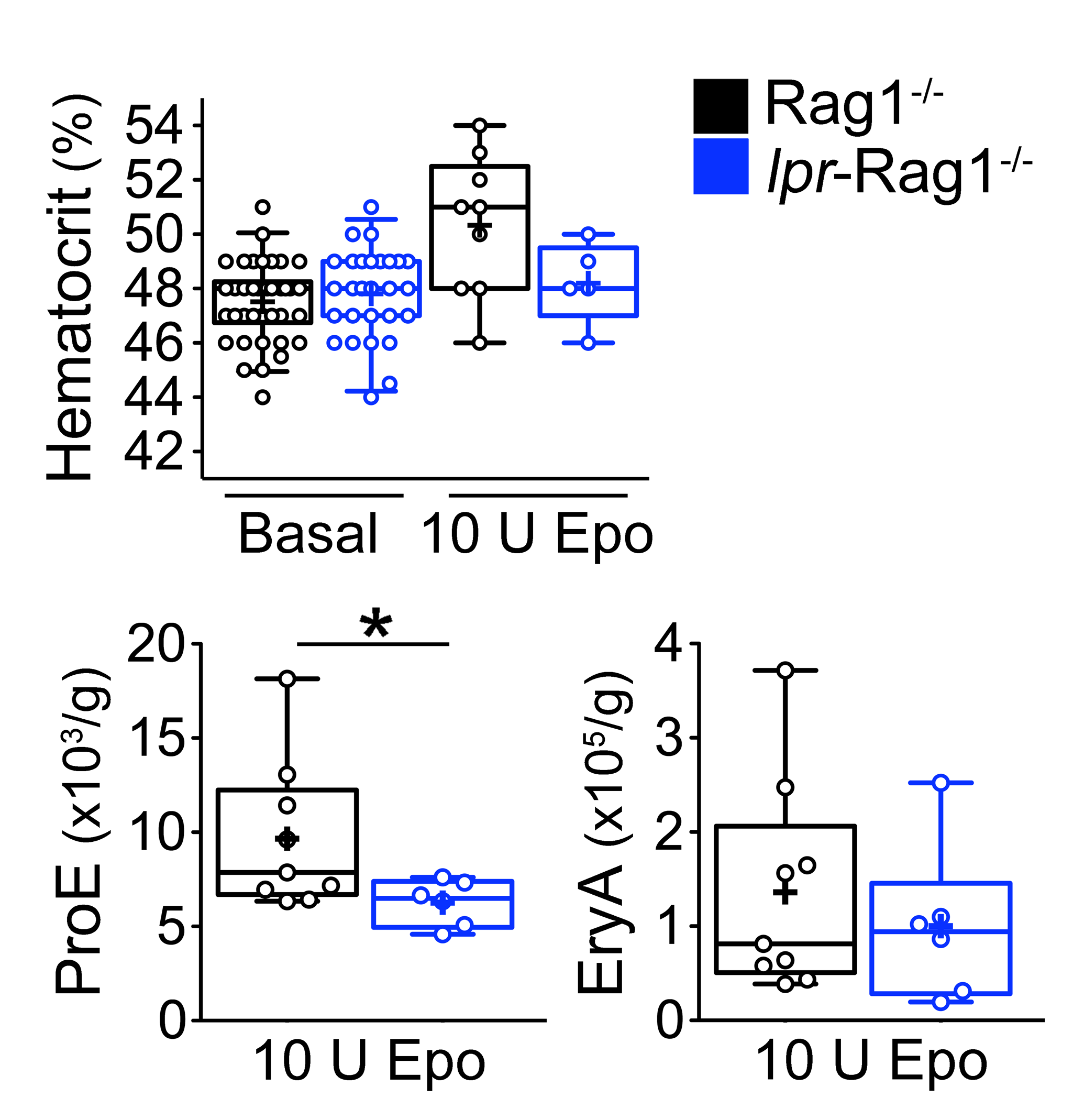

Supplement: Figure S3 — Associated with Fig 6: Delayed response to Epo injection in lpr-Rag1−/− mice. Female mice were injected with 10 U of Epo subcutaneously. Hematocrit, ProE and EryA progenitors were measured on day 3 post injection. *p<0.05 (two-tailed t test, unequal variance). Basal hematocrit values are for lpr-Rag1−/− females. (TIF) [file pone.0021192.s003.tif]

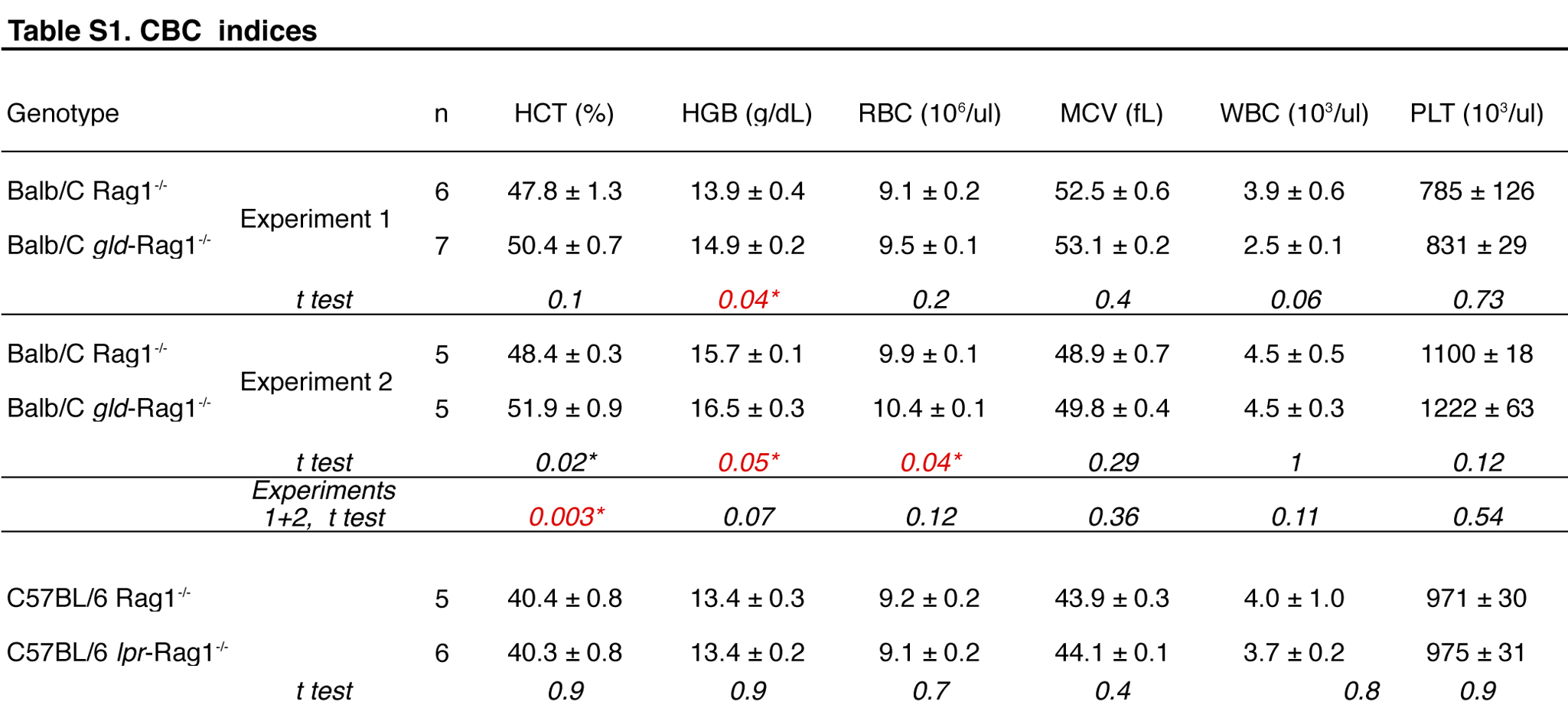

Supplement: Table S1 — Associated with Fig 3: Complete blood counts (CBC) for the indicated mouse strains. Number of mice used for each strain in each experiment is indicated in parentheses. HCT = hematocrit. HGB = hemoglobin. RBC = red blood cells. MCV = mean corpuscular volume. WBC = white blood cells. PLT = platelets. All data are mean ±sem. t test (unequal variance) p values are indicated. (TIF) [file pone.0021192.s004.tif]
