# Supplementary material for: Negative Autoregulation by Fas Stabilizes Adult Erythropoiesis and Accelerates Its Stress Response
Source: PLoS One. 2011 Jul 8;6(7):e21192. doi: 10.1371/journal.pone.0021192 (PMC3132744; doi:10.1371/journal.pone.0021192)
Supplement: Text S1 — Supplementary Materials and Methods. (DOCX) [file pone.0021192.s005.docx]

## Text S1: Supplementary Materials and Methods

#### Mice

Balb/C mice were purchased from Taconic, C57BL/6J, strains were purchased from the Jackson Laboratories (Bar Harbor, ME). MyD88^-/-^ mice were kindly provided to us by Dr. Egil Lien (University of Massachusetts Medical School, Worcester, MA). B6.MRL-Fas^lpr^/J (Jackson Laboratories stock # 000482) were crossed with B6.129S7-Rag1^tm1Mom^/J (stock # 002216) to obtain homozygous double-mutant lpr-Rag1^-/-^ mice (C57BL/6J background). The homozygous double mutant gld-Rag1^-/-^ mice (Balb/C background) were obtained by crossing CPt.C3-Fasl^gld^/J (Jackson Laboratories, stock # 002932) with C.129S7(B6)-Rag1^tm1Mom^/J (Jackson Laboratories stock # 003145). Genotyping was performed by PCR according to Jackson Laboratories protocols. In all experiments, lpr-Rag1^-/-^ and gld-Rag1^-/-^ mice were compared to their respective Rag1^-/-^ controls. Mice were used at 10 to 14 weeks of age. All experiments with mice were conducted in accordance with an animal protocol approved by the University of Massachusetts Medical School IACUC committee.

#### Erythropoietic stress

Recombinant human erythropoietin (Epoetin alfa, Amgen, Thousand Oaks, CA) was injected at the indicated doses subcutaneously in a total volume of 150μl in sterile isotonic saline. Human purified Fas:Fc chimeric fusion protein (BD Biosciences, San Diego, CA) was injected at 100 μg/ per mouse, intraperitoneally. Reduced atmospheric oxygen treatment was conducted using the BioSpherix A-chamber (BioSpherix, Lacona, NY) for the indicated time periods. Hypoxia was achieved by displacing oxygen with nitrogen at normal atmospheric pressure. Temperature, humidity and carbon dioxide readings were monitored.

#### Labeling of erythroid progenitors in fresh tissue for flow cytometry

Antibody staining and flow cytometry were performed as described (Liu et al., 2006). Briefly, freshly isolated spleen and bone-marrow cells were suspended in phosphate-buffered saline with 5% fetal bovine serum and immunostained in the presence of blocking rabbit or mouse immunoglobulin (Jackson ImmunoResearch Laboratories, West Grove, PA). Dead cells were excluded using DAPI (Roche, Indianapolis, IN) or 7-AAD (BD Biosciences) viability dye. The following antibodies and reagents were used: PE- or FITC-rat anti-mouse CD71 (C2 clone), PE- or APC-rat anti-mouse Ter119, biotin-hamster anti-mouse Fas (Jo2 clone) or FasL (MFL3 clone) (BD Biosciences). Streptavidin-APC (Invitrogen, Carlsbad, CA) was used to amplify the biotin signal. Alexa Fluor 350-Annexin V (Invitrogen) staining was performed according to the manufacturer’s instructions. LSRII (BD Biosciences) flow cytometer was used for sample detection. All data were analyzed using FlowJo software (Tree Star, Ashland, OR).

#### Reticulocyte detection

Reticulocytes were detected as a DNA-negative, RNA-positive cell population in blood, as described (McGrath et al., 2008). EDTA-preserved whole blood was stained at room temperature in PBS with RNA/DNA dye Thiazole Orange (Sigma-Aldrich, St. Louis, MO), followed by staining with DNA dye DRAQ5 (BioStatus, Shepshed, United Kingdom). Data were collected using the 488nm and the 633nm lasers (FITC and APC (670/40 filter) channels, respectively) on LSRII (BD Biosciences) flow cytometer and analyzed using FlowJo software (Tree Star).

#### Peripheral blood analysis

Hematocrit was determined using the CritSpin Microhematocrit centrifuge kit (Iris Sample Processing, Westwood, MA). For Complete Blood Count (CBC), whole blood was collected in EDTA collection tubes and processed on Heska Hematology Analyzer (Heska, Loveland, CO).

**CFU-e colony assays**

Freshly isolated spleen and bone-marrow cells were manually counted, and cells were plated for tissue culture in M3231 Methocult® methylcellulose media (StemCell Technologies, Vancouver, Canada) supplemented with 2 U/ml rh-Epo (Amgen). On day 3, plates were stained with 3,3'-Diaminobenzidine (Sigma-Aldrich) and scored for erythroid colonies. Results are expressed as total CFU-e colonies per 10^6^ live plated cells.

**Epo ELISA**

Enzyme Linked Immunosorbent Assay (ELISA) for mouse Epo was performed according to the manufacturer’s instructions (Quantikine ELISA, R&D Systems, Minneapolis, MN). EnVision 2102 Multilabel Reader (Perkin Elmer, Waltham, MA) was used to quantify fluorescence. Data was converted into mU/ml by multiplying pg/ml value by 129,000 IU/mg (International Standard for fully glycosylated Epo protein (Jelkmann, 1992)).

**Statistical analyses**

Statistical analysis of basal state data was performed using PASW (SPSS, Chicago, IL) and SAS (SAS, Cary, NC) statistical software. Data was log-transformed and residuals were tested for normality by Kolmogorov-Smirnov test (performed in PASW). Log-transformed data was then analyzed by general linear mixed model ANOVA with gender and genotype as fixed effects and experiment as the random effect (performed in SAS). Unadjusted p-values (p<0.05 considered significant) were reported in the figures for each gender-genotype group.

F probability distribution test (F test) was performed on residuals derived from untransformed data. Experiment, gender and genotype were taken into account for deriving the residuals (performed via PASW) prior to F test. Similar, or even more significant, F test p-values were obtained via a traditional F test on raw pooled data.
