# Supplementary material for: Negative Autoregulation by Fas Stabilizes Adult Erythropoiesis and Accelerates Its Stress Response
Source: PLoS One. 2011 Jul 8;6(7):e21192. doi: 10.1371/journal.pone.0021192 (PMC3132744; doi:10.1371/journal.pone.0021192)
Supplement: Text S2 — Regulation of the EryA progenitor pool by Fas. (DOCX) [file pone.0021192.s006.docx]

### Text S2: Regulation of the EryA progenitor pool by Fas

The data in Figure 2A (left panel) can be fitted by a model in which Fas acts as a negative autoregulator of the EryA pool (Fig 2B).

Let the number of EryA cells within the erythroblastic island be ‘*A*’. *‘A’* is expressed relative to the basal frequency of EryA within the erythroblastic island, (measured experimentally as the fraction of Ter119^+^ cells that are ‘EryA’ in the basal state).

‘*A*’ is the result of three principal factors (Fig 2B):

- a continuous input from earlier progenitors, *β*;
- a continuous output, *αA*, into more differentiated progenitor subsets;
- Fas-dependent loss through cell death.

The probability of Fas-mediated cell death is proportional to the probability of a Fas-positive EryA cell encountering an EryA cell that expresses FasL, at sufficiently close quarters to result in activation of the Fas receptor. If the fraction of EryA cells that expresses Fas is denoted by *‘F’*, then the frequency of Fas-positive EryA cells in the erythroblastic island is *‘AF’*; similarly, if the frequency of FasL expression amongst EryA is *‘L’*, the frequency of FasL-positive EryA cells in the island is *‘AL’*. The probability of this encouter is therefore proportional to
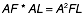
. However, since nearly all EryA cells express FasL, and since the change in FasL expression in response to Epo is relatively small, we can substitute
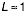
. The probability of Fas-mediated cell death is therefore proportional to *‘A^2^F’,* a term that varies with the square of the frequency of EryA in the erythroblastic island, for any given level of Fas.

This model allows us to predict how the frequency of EryA changes with Fas expression in response to Epo. Thus, change in EryA pool over time,
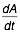
, is given by:


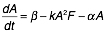


where ‘*k*’ is a proportionality constant. When a new steady state is attained between days 2 and 3 following an Epo injection (Liu et al), there is no net change in ‘*A*’, making
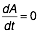
. Therefore, equation becomes:


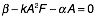


For a given constant value of *β, α* and *F*, the corresponding value of *‘A’* may be found by solving equation as a simple quadratic equation:


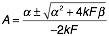


Epo levels change with erythropoietic stress, suppressing *F*. We assume that the input *β* and the output coefficient, *α*, remain relatively constant in spite of changes in the level of Epo. The inverse relationship between EryA (‘*A’, expressed relative to the basal frequency of EryA within Ter119^+^ cells*) and the number of Fas^+^ EryA (‘*F’, expressed relative to the frequency of Fas^+^ cells within EryA cells*) in Fig 2A is fitted well by a hyperbolic curve that represents the (positive) solutions for *‘A’* in equation , for different steady-state levels of Fas *(‘F*’) (R^2^=0.89).

The initial fitted constants were *β*=1.3, *α*=0, *k*=0.5. R^2^=0.88.

The zero value fitted by the ‘solver’ software for *α* reflects its low actual value relative to *β*, with the result that the curve fitting for equation is relatively insensitive to small changes in *α* around zero. To find the actual value for *α*:

As F
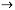
0, then, from equation ,


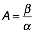


Experimentally, we know that under these circumstances, ‘*a’* is in the order of 30 to 60-fold the basal EryA pool (This increase is the measured expansion of EryA. Figure 4A and main text). From equation , and from the value for *β* obtained by the curve fitting, this gives a value for *α* between 0.02 and 0.04. Substituting these values in the equation describing the curve in Figure 2A does not substantially alter the curve and its fitness to the data (R^2^ remains 0.89).
